# Responsiveness level and its effect on services quality from the viewpoints of the older adults hospitalized during COVID-19 pandemic

**DOI:** 10.1186/s12877-022-03344-5

**Published:** 2022-08-09

**Authors:** Ali Reza Yusefi, Esmat Rezabeigi Davarani, Salman Daneshi, Misagh Bastani, Gholamhossein Mehralian, Peivand Bastani

**Affiliations:** 1 Department of Public Health, School of Health, Jiroft University of Medical Sciences, Jiroft, Iran; 2 Health in Disasters and Emergencies Research Center, Institute for Future Studies in Health, Kerman University of Medical Sciences, Kerman, Iran; 3 Shooshtari Hospital, Shiraz University of Medical Sciences, Shiraz, Iran; 4 Nottingham Business School, Nottingham, UK; 5 Faculty of Health and Behavioral Sciences, School of Dentistry, University of Queensland, Brisbane, Australia

**Keywords:** Responsiveness, Quality of Services, Aged, Hospital

## Abstract

**Background:**

Aging is a sensitive period of life. Attention to the needs of this stage is considered a social necessity. This study is conducted to investigate the responsiveness level and its effect on service quality from the hospitalized older adults’ viewpoints during the COVID-19 pandemic in the south of Iran.

**Methods:**

It was a cross-sectional descriptive-analytic study that was conducted on 386 old patients. The study instrument was a standard questionnaire that includes three sections of demographic information, World Health Organization Responsiveness, and SERVQUAL. Data were analyzed applying descriptive and inferential statistics the same as Independent T-test, ANOVA, Pearson correlation, and multiple linear regression.

**Results:**

The mean levels of responsiveness and service quality were 90.72 ± 9.38 (from 160) and 68.01 ± 8.51 (from 110) respectively. This indicates the average level of these variables from the old patients’ viewpoints. There was a significant positive correlation between responsiveness and service quality (*r* = 0.585). According to the results of multiple linear regression, the dimensions of communication, dignity, prompt attention, primary facilities, social support, information confidentiality, right to choose, and autonomy were identified as the predictors of service quality.

**Conclusion:**

The average level of responsiveness and service quality perceived from the old patients’ viewpoints during the COVID-19 pandemic can be considered a necessity for supportive planning among the older adults. Meanwhile, according to the impacts of responsiveness on service quality, educational programs are recommended to promote the level of healthcare providers’ responsiveness.

## Background

The first case of new coronavirus (COVID-19) was reported in Wuhan, China and then it was spread all over the world [1]. Following the rapid spread of the disease, on March 11, 2020, the World Health Organization (WHO) has announced COVID-19 as a pandemic [2]. Until February 20, 2022, more than 400 million confirmed positive cases and more than 5 million cases of death were estimated. Iran as a developing country in the Middle East has experienced more than 6 million cases of morbidity and more than 130,000 mortality cases [3]. After confirmation of the first positive case of COVID-19 in February 2020 in Qom, Iran, 41 referral hospitals along with 168 educational hospitals and health centers with the high potentiality of emergency evacuation have been allocated to the COVID-19 related services. Meanwhile, more than 5000 urban health stations and 5000 rural comprehensive health centers were allocated to tracing and following the suspected and positive cases [4].

In this condition, the world population moves toward aging [5]. Statistics indicate the changes in the age pyramid of the population [6]. Developing countries, the same as the developed ones, encounter the aging phenomenon [7]. It is expected that more than Asian countries will have a population with at least a quarter more than 60 years till 2050 [8]. In Iran, the results of a survey indicate an incredible increase in the older adults with 60 years old and more from 5.7% in 2011 to 6.1% in 2016 [9]. Continuing such a trend, in a near future, Iran may face an aging phenomenon [10]. The beginning aging period is accompanied with physical and mental complications. According to the studies, people with contextual diseases and older adults are among the high-risk groups against COVID-19 [11, 12]. Other study results have indicated that age can be considered as a mortality risk factor related to COVID-19. For instance, results of a study in China [13], Italy [14], France [15], and the USA [16] have emphasized the increased rate of mortality as a result of hospitalized cases of COVID-19 among the older adults.

Along with communicable diseases, aging is accompanied with more prevalence of chronic and non-communicable diseases [17]. Almost 75% of those people with 60 years and more, at least suffer from one kind of chronic disease and almost 50% of them have two simultaneous diseases [18]. So, it is expected that a huge amount of hospitalized patients is among old people [19]. In such a condition, promoting the health status of older adults hospitalized is considered as one of the most challenges for health care systems [20]. Physiological changes, psychological and social needs of the old people have led to different quantity and quality of health cares for this group comparing the other patients [21]. In this regard, evidence indicates that average length of stay (ALS) among hospitalized old patients is about 10 times more than younger groups [22]. So, the appropriate identification of the needs and suitable quality of the services for this group is an inevitable necessity [23]. Without this consideration, the healthcare systems may tolerate high costs along with patients’ pain and discomfort [24].

On the other hand, high-quality health services for old patients depend on formal and informal support and effective responsiveness [25]. In the hospital environment, keeping and promoting the responsiveness to old patients is among one of the main cores of health care and can be considered as an important factor for increasing patient satisfaction, appropriate therapeutic outcomes, and patient treatment compliance and adherence [26]. At the same time, responsiveness is considered as one of the final outcomes of every health system [27]. This concept is among one of the frameworks for evaluating the quality of non-clinical health care services that is presented by WHO in 2000. According to this framework, for the evaluation of non-clinical dimensions of the quality or “responsiveness of the health system to non-medical needs”, eight main areas are considered via two categories. The first category is respect to the people’s rights. This includes dignity, autonomy, information confidentiality, and communication. The second category is alluded to the customers. This includes the right to choose, prompt attention, primary facilities, and social support [28, 29]. The evidence of developing countries has shown that the level of responsiveness is accompanied with positive outcomes in the process of service delivery to the patients in a way that the satisfied patients have more compliance of the treatment recommendations, give more information about their health cares and continue their health services [30–32]. Moreover, responsiveness can lead to an increase in the sense of comfort and better collaboration of the patients in utilizing health care services and finally promoting their health [33, 34]. The results of some studies indicate different levels of responsiveness and the state of its dimensions. So that in some studies conducted in Iran reported an average level of responsiveness [35–37], and a study conducted in Nigeria reported an appropriate level [38] of responsiveness. Also, the status of each dimension of responsiveness in previous studies has been stated differently [39–48].

More than responsiveness, assessing and managing the quality of services for health centers and hospitals. Quality services can save resources as well as create a satisfactory environment [49]. In addition, achieving the desired quality of health services leads to patient satisfaction, effectiveness of service providers, increasing employee morale and reducing service costs [50, 51]. One of the models developed in measuring service quality is the SERQUAL. This model tries to measure the quality of services where service quality is a necessity for understanding the customer and his expectations [52]. According to this model, service quality can be measured in five dimensions: Tangibles (the appearance of physical facilities, equipment, personnel, and communication materials), Responsiveness (the willingness to help customers and to provide prompt service), Assurance (the knowledge and courtesy of employees and their ability to convey trust and confidence), Reliability (the ability to perform the promised service dependably and accurately), and Empathy (the provision of caring, individualized attention to the customer) are examined [53]. Regarding the quality of hospital services, the findings of previous studies before and after the outbreak of COVID-19 have reported different levels of service quality and its dimensions status [54–64].

Sufficient information about perceived content from the service providers can help the organizations in identifying the contexts and dimensions of prior competition and avoiding resources waste [65, 66]. Benefiting from the health service users’ viewpoints also can lead to evaluating and promoting the health centers’ performance. Thus, investigating the status of responsiveness and service quality of the health care providers the same as hospitals are among the necessities for the health systems. This study is aimed to determine the responsiveness of old patients and its effect on the quality of the provided services during COVID-19 pandemic. The present results can pave the way for the health policy makers and health care providers for better provision of the services and more appropriate outcomes.

## Theory and hypothesis development

Responsiveness is an important approach to measuring the quality of services that is based on utility theory [67]. This theory refers to the measurement of preferences over a set of services [67]. In addition, the human rights law emphasizes responsiveness as one of the characteristics of health systems and their subordinate organizations, a very important category [68, 69]. The positive effects of responsiveness can facilitate organizational goals. In contrast, the weak responsiveness of the health organizations like hospitals may be accompanied by destructive effects on the quality of services [70]. For instance, Khorasani Zavareh et al. have shown that the effective factors on the quality of services include the personnel’s behavior, skills, and efficiency, the length of service delivery processes, appropriate directing of the patients, and, respecting to the patients’ rights [71]. Atinga et al. have also mentioned five factors of support and responsiveness, methods of care, equipment, environment, and, waiting time as the main predictors of service quality in Ghanaian hospitals [72]. A large body of scholarly research has been conducted to investigate the relationship between responsiveness and perceived service quality by patients. Nambisan et al. confirmed that responsiveness positively affects patients’ perception of service quality [73]. In another study in Croatia, the authors highlighted to render quality services, hospital settings should pay more attention to responsiveness compared to other issues [74]. It is also found that the non-responsiveness of healthcare centers negatively affects the perceived quality by patients [75, 76].

In addition, some studies indicate that responsiveness has been significantly associated with some demographic variables such as marital status [77]. Also, the relationship between service quality and variables such as gender [78–80] and education level [78, 81–84] has been reported in some other studies.

According to the theoretical framework of reviews, debates, and various adaptations regarding responsiveness and service quality [70–84], we propose:

- H1. Responsiveness dimensions have positive effects on the perceived quality by COVID-19 patients.
- H2. The means score of responsiveness varies significantly in terms of demographic variables.
- H3. The means score of service quality varies significantly in terms of demographic variables.

## Methods

### Participants

The study population consists of those hospitalized patients in therapeutic-educational hospitals affiliated with Shiraz University of Medical Sciences (*N*_=_8 hospitals) that were in one of the inpatient wards at the beginning of the study receiving the healthcare services. Considering the following formula [85] and the correlation between responsiveness and quality services on the basis of a pilot study in Iran (*r* = 0.2) with 95% confidence level and β = 0.1, the sample size was estimated to be at least 259. In order to increase the accuracy and avoid bias as a result of sample attrition, 386 participants were included.

1 $$n= {\left[\left({Z}_{1-\alpha /2}+{Z}_{1-\beta }\right)/\mathrm{ W} \right]}^{2}+3$$

In formula 1, W is calculated using the following formula:

2 $$W= 1/2 \;1n\left(1+\mathrm{r}/1-\mathrm{r}\right)$$

In formula 2, *r* is the estimated correlation coefficient between responsiveness and service quality in a pilot study in Iran [86].

The distribution of the questionnaires among these 386 participants in the hospitals was occurred as a proportion to the size of the population of each of the studied hospitals. For this purpose, after referring to each hospital, a total of old patients (60 years old and more) who were hospitalized in different inpatient wards was identified. After calculating the total number of hospitalized old patients, in order to distribution of 386 questionnaires, a proportional sampling related to the population was used. In each hospital, the number of included participants was allocated appropriately according to the number of hospitalized patients in that ward.

The inclusion criteria include voluntary consent, age 60 years or more, and the ability to talk. According to WHO, age 60 years old and more in developing countries were defined as the base of aging [87]. As cognitive disorders like Delirium and major and minor neurocognitive disorders are considered among those psychological disorders that affect cognitive abilities (learning, memory, perception, and concentration) [88], those patients with the above complications were excluded. For this purpose, with the help of the head nurse and physician, the patients` medical records were reviewed and patients with the above problems were identified.

### Instruments

The data collection tool was a three-section questionnaire. The first section consists of the old patients’ demographic information including age, gender, marital status, education level, and income level. The second section then includes a standard questionnaire of responsiveness designed by the World Health Organization (WHO). In this study according to the research objectives, the health system responsiveness model was applied among all the survey questionnaires designed by WHO [89]. In this section, 32 items were considered in 8 dimensions of “prompt attention” (3 items), ‘communication” (8 items), “dignity” (8 items), “autonomy” (4 items), “information confidentiality” (2 items), “right to choose” (2 items), “primary facilities” (3 items) and, “social support” (2 items). In this section, a five scale of Likert was applied for assessing the responsiveness (1–5 for very low, low, medium, high, and very high respectively). According to the score domain (32 to 160), responsiveness status from the old patients’ viewpoints according to the score domain was defined as 32–57 (very weak responsiveness), 58–83 (weak responsiveness), 84 to 109 (average responsiveness), 110–135 (good responsiveness) and 136–160 (very good responsiveness). The scoring was divided into 5 modes according to the WHO approach [67].

Finally, in the third section of the questionnaire, in order to assess the quality of services from the old patients’ viewpoints, the SERVQUAL questionnaire was applied [90]. This questionnaire includes 22 items in 5 dimensions as follows: “Tangibles” (4 items), “Responsiveness” (4 items), “Assurance” (4 items), “Reliability” (5 items) and, “Empathy” (5 items). For this part, a 5-scale Likert was used from 1 to 5 for very weak, weak, average, good, and very good respectively. According to the score domain (22-110), the service quality status was evaluated from the old patients’ viewpoints as the score domain of 22–39 was allocated to very poor service quality, 40–57 was related to poor quality services, and score domains of 58 to 75, 76 to 93 and 94 to 110 were allocated to average, good and very good quality of services respectively. The points were classified into 5 mentioned modes according to the SERVQUAL Parasuraman model [91]. WHO responsiveness questionnaire was translated into Persian and its reliability and validity were approved by Rashidian et al. [92]. The reliability of the questionnaire was assessed by test–retest with 30 pre-test cases in the interval of one week for the post-test. Cronbach α was estimated as 0.91. Cronbach α for the questionnaire dimensions were between 0.76 to 0.89.

The validity and reliability of the SERVQUAL questionnaire were also approved in the previous studies [90, 93]. In order to assess the reliability of the instrument in this study, an internal compatibility analysis was applied. Cronbach α was estimated from 0.84 to 0.86 for the dimensions and 0.89 for the total of 22 questions.

### Procedure and statistical analysis:

It was a cross-sectional descriptive-analytic study that was conducted from June to October 2021. Regarding the research procedures, two of the researchers (ERD and MB) referred to the concerned hospitals on different weekdays in morning, evening, and night shifts and distributed questionnaires, and collected the required information. Individuals willingly took part in the study and filled out the questionnaire. After obtaining the necessary permits from the Shiraz University of Medical Sciences and explaining the objectives of the project to the participants, the confidentiality of information was emphasized, and their verbal satisfaction was obtained. Questionnaires were then distributed among the patients. Questionnaires were completed by the patients; however, some patients asked the research team (ERD and MB) to help them fill out the survey. After registering the participants’ answers to the questionnaires’ sheets, data was imported to IBM SPSS Statistics 23 software and analyzed applying descriptive statistics as well as inferential statistics (Independent T-test, ANOVA, Pearson correlation, and multiple linear regression ones) at the significant level of 0.05. Since Pearson correlation coefficient is a value between -1 to + 1, so the coefficient of 0 to 0.29, 0.30 to 0.69, and 0.70 to 1 indicate a weak, medium and strong positive correlation, respectively [94].

In order to investigate the correlation between the main variables (responsiveness and quality of services) and their dimensions, Pearson correlation was used. Pearson correlation was also applied to test the association between the main variables and the patients’ age.

More than correlation, for comparing the mean difference between responsiveness and quality of services according to the patients’ gender, Independent t-test was used. ANOVA was also applied for the mean difference of the main variables according to the other demographic variables as marital status, education level, and income level. In ANOVA test, in case of significant differences in the mean of variables based on different groups, Scheffe post hoc test was used. This test is a good way to compare the means of groups with unequal volume [95].

The multiple linear regression was used to investigate the effect of responsiveness different dimensions (as the independent variable) on the service quality (dependent variable). In the regression model, R-squared shows what percentage of the dependent variable changes are explained by the independent variables. The value of this index is between zero and one and if it is more than 0.6, it shows that the independent variables have been able to explain the changes of the dependent variable to a large extent [96]. In addition, one of the presuppositions of multiple linear regression is the absence of collinearity or correlation between independent variables. VIF index was used to check for non-alignment. According to statistical logic, if the VIF is greater than 10, then alignment is possible [97].

## Results

The average age of the participants was 68.62 ± 5.18 years and 24.61% of them were among the group of 65–69 years. Among the participants, 33.94% were identified as the elementary school level. Most of the participants were male (53.89%), married (70.47%) with an income level of 10–20 million Rials Iranian currency (38.997–77,954$) (50.52%).

Regarding H2 and H3, the responsiveness level from the old patients’ perspective was significantly different based on gender and marital status. The mean score of responsiveness from the men`s perspective (94.36 ± 7.43) was higher than the women’s (87.09 ± 8.86). According to the Scheffe post hoc analysis, the mean score of responsiveness was significantly different among single and married groups (*p* = 0.04). The mean score of responsiveness among married groups shows 5.14 units increase than the single ones. The quality of services from the old patients’ viewpoints was subject to gender and level of education. The mean score of service quality was higher from the men’s perspective (74.19 ± 9.42) than the women’s (61.83 ± 7.29). Scheffe’s post hoc test shows that the mean score of service quality among old patients’ with the education level of elementary school and those who have diploma were significantly different (*p* = 0.04) as well as the group with Bsc and higher education (*p* = 0.03). In this regard, the mean score of service quality among those in elementary school has increased 4.73 units than the group with diploma and 5.01 units increase were demonstrated compared with those who have BSc or higher degrees (Table 1).

**Table 1:** Characteristics of study participants (*n* = 386)

**Variable**	**Category**	**Frequency (%)**	***P*-value** Responsiveness Service quality
**Age (year)**^a^	60–64	74 (19.17)	0.4 0.6
	65–69	95 (24.61)	
	70–74	72 (18.65)	
	75–79	56 (14.51)	
	80 +	89 (23.06)	
**Gender**	Male	208 (53.89)	**0.01** **0.02**
	Female	178 (46.11)	
**Marital Status**	Single	69 (17.87)	**0.03** 0.1
	Married	272 (70.47)	
	Divorced	13 (3.37)	
	Widow	32 (8.29)	
**Level of Education**	Unable to Read and Write	65 (16.84)	0.08 **0.04**
	Reading and Writing	86 (22.28)	
	Elementary School	131 (33.94)	
	Diploma	56 (14.51)	
	BSc and higher	48 (12.43)	
**Income Level (Rials)**	< 10,000,000	64 (16.58)	0.09 0.1
	10,000,000–20,000,000	195 (50.52)	
	20,000,001–30,000,000	74 (19.17)	
	> 30,000,000	53 (13.73)	
**Total**	–––	386 (100)	–-

^a^WHO World Standard Population Distribution (%), based on world average population between 2000–2025 [98]

The total mean score of responsiveness was 90.72 ± 9.38. According to the applied Likert scale, it indicates the average level of responsiveness according to the studied old patients. According to the results, from the viewpoints of 50.37% of the studied old patients, responsiveness was reported at an average level. Moreover, among the responsiveness dimensions, 52.07% of the studied old patients have reported “communication” at a weak level (Table 2).

**Table 2:** The mean score of responsiveness and its determinants

	**Dimension**	**Score domain**	**Mean ± SD**	**%**				
				Very weak	Weak	Average	Good	Very good
**Responsiveness**	Prompt Attention	3–15	9.27 ± 3.14	2.33	8.03	58.55	28.24	2.85
	Dignity	8–40	21.81 ± 5.81	3.63	34.71	47.67	11.92	2.07
	Communication	8–40	19.62 ± 4.59	6.48	52.07	37.04	3.11	1.8
	Autonomy	4–20	10.56 ± 3.22	2.58	28.25	59.33	8.29	1.55
	Primary facilities	3–15	11.09 ± 3.36	1.55	3.11	49.85	39.01	6.48
	Information Confidentiality	2–10	6.49 ± 2.61	3.37	4.4	48.45	35.75	8.03
	Right to choose	2–10	5.76 ± 2.54	4.67	10.7	50.85	28.34	5.44
	Social Support	2–10	6.12 ± 2.43	2.85	12.95	61.92	21.24	1.04
	**Total**	**32–160**	**90.72 ± 9.38**	**3.6**	**20.78**	**50.37**	**21.38**	**3.87**

The mean score of service quality was 68.01 ± 8.51. It was indicated an average level of service quality according to the studied old patients’ viewpoints. According to the results, 55.85% of the studied old patients were reported an average level of service quality. Meanwhile, among the service quality dimensions, 22.28% of the old patients, have reported “Assurance” dimension in a weak level (Table 3).

**Table 3:** The mean score of service quality and its determinants

	**Dimension**	**Score domain**	**Mean ± SD**	**%**				
				Very weak	Weak	Average	Good	Very good
Service Quality	Tangibles	4–20	13.09 ± 2.86	2.07	4.15	52.07	37.05	4.66
	Responsiveness	4–20	12.26 ± 4.46	3.89	10.08	60.88	22.54	1.88
	Assurance	4–20	11.37 ± 4.83	8.55	22.28	50.78	16.84	1.55
	Reliability	5.25	16.18 ± 5.12	3.11	4.92	60.1	29.02	2.85
	Empathy	5.25	15.11 ± 4.79	3.37	16.58	55.44	22.28	2.33
	**Total**	**22–110**	**68.01 ± 8.51**	**4.2**	**11.76**	**55.85**	**25.55**	**2.64**

There was a statistically significant positive correlation between all dimensions of responsiveness and service quality (*p* < 0.05). Further, other results illustrated in Table 4 show that there was a significant correlation between service quality and responsiveness from the old patients` viewpoints (*r* = 0.585, *p* < 0.001). Given the value of r, this positive correlation was at a moderate level.

**Table 4:** The correlations between responsiveness and quality of services and their dimensions from the old patients’ viewpoints

	**Responsiveness Dimensions**^a^									
Service Quality Dimensions^a^		PA	DI	CM	AU	PF	IC	RC	SS	**TRS**
	TA	0.340^**^	0.421^***^	0.501^***^	0.432^***^	0.721^****^	0.386^**^	0.378^**^	0.496^***^	0.495^****^
	AS	0.694^****^	0.628^****^	0.701^****^	0.612^****^	0.414^***^	0.636^****^	0.644^***^	0.610^****^	0.618^****^
	RS	0.702^****^	0.691^****^	0.681^****^	0.673^****^	0.669^***^	0.651^****^	0.683^****^	0.672^****^	0.677^****^
	RE	0.571^****^	0.558^****^	0.563^****^	0.467^***^	0.481^***^	0.546^****^	0.435^***^	0.449^***^	0.508^****^
	EM	0.694^****^	0.711^****^	0.685^****^	0.586^****^	0.679^****^	0.577^****^	0.561^****^	0.688^****^	0.647^****^
	**TSQ**	0.601^****^	0.610^****^	0.631^****^	0.543^****^	0.597^****^	0.562^****^	0.557^****^	0.588^****^	0.585^****^

^a^*PA* Prompt Attention, *DI* Dignity, *CM* Communication, *AU* Autonomy, *PF* Primary Facilities, *IC* Information Confidentiality, *RC* Right to choose, *SS* Social Support, *TRS* Total Responsiveness, *TA* Tangibles, *AS* Assurance, *RS* Responsiveness, *RE* Reliability, *EM* Empathy, *TSQ* Total Service Quality

^**^p < 0.05,

^***^p < 0.01,

^****^p < 0.001

In order to identify the simultaneous effects of the responsiveness dimensions on service quality according to the old patients’ perspective, multiple linear regression was used. For this purpose, first the assumptions of linear regression were studied that showed normal residues, inequality of variance of residues, lack of serial correlation among residues and, lack of collinearity among independent variables. The VIF value for all variables was estimated to be less than 3, indicating a lack of correlation and lack of alignment between the independent variables.

Results of the multiple linear regression show that the significant variables in the model, which were determined using the Enter method were communication, dignity, prompt attention, primary facilities, social supports, information confidentiality, right to choose, and autonomy respectively. The related β amounts of effective variables that indicate the priority of affecting the quality of services were presented in Table 5. The results of the statistical test also have shown that the coefficient of determination for the processed model (R-squared) was 0.59 which means 59% of the variation in the service quality scores can be explained by the model variables. Given that the value of R-squared is close to 0.6, the dimensions of responsiveness as independent variables have been able to largely explain the changes in service quality as a dependent variable.

**Table 5:** The effect of responsiveness dimensions on service quality simultaneously

Variables	Unstandardized coefficients		Standardized coefficient β	t-statistics	*P*-Value
	**Coefficient β**	**Std. Error**			
(Constant)	6.304	1.203	–-	2.846	0.004
Communication	0.443	0.044	0.393	3.682	< 0.001^*^
Dignity	0.427	0.057	0.384	3.631	< 0.001^*^
Prompt Attention	0.424	0.063	0.377	3.596	< 0.001^*^
Primary Facilities	0.418	0.069	0.368	3.487	< 0.001^*^
Social Support	0.411	0.072	0.361	3.446	< 0.001^*^
Information Confidentiality	0.393	0.083	0.343	2.953	0.001^*^
Right to choose	0.387	0.091	0.337	2.861	0.001^*^
Autonomy	0.369	0.097	0.319	2.508	0.002^*^

Results of multiple linear regression shows the hypothesis 1 is confirmed. This is that responsiveness elements simultaneously have positive effects on the perceived quality by COVID-19 patients.

## Discussion

According to the results of this study, from the perspective of older adults, the responsiveness was average. In line with this finding, some studies conducted in Iran have also shown the average level of hospital responsiveness from patients [35–37]. However, it contradicted the findings of a study by Adekanye et al. [38] in Nigerian hospitals, which showed a relatively good level of responsiveness. It seems that one of the reasons for this discrepancy could be the sample and the different settings of the two studies. In addition, the prevalence of COVID-19 at the time of the present study may have affected patients’ perceptions of responsiveness.

In the present study, 39.01% and 35.75% of the participants, respectively have evaluated the primary facilities and information confidentiality dimensions at a good level. Similar to the present results, in a study in Tehran, Iran, most of the patients infected with COVID-19 were satisfied from the service delivered and facilities [39]. Wu et al. in their study in England have also reported that 94% of those COVID-19 patients hospitalized in the inpatient wards had a sense of security and 93% of them have declared that their privacy was preserved during hospitalization [40]. Nemati et al. have also indicated that most of the patients were satisfied from preserving their privacy during physical exams and therapeutic procedures [41]. It seems that preparing primary facilities and preserving the patients’ privacy and confidentiality can lead to a sense of comfort and satisfaction of the received services [39, 42].

In this study in a responsiveness area, 52.07% of the old patients have evaluated the communication dimension at a weak level. Results of Gohari et al. in 5 large educational hospitals in Iran have shown that among responsiveness dimensions, “communication” and “informing” have the least mean scores [43]. In another study in Taiwan that was conducted according to different dimensions of responsiveness proposed by WHO, the ability to communicate between the treatment team and the patients and fulfilling the medical ethics were among the most concerns of the patients [44]. According to different studies, effective communication among Physicians, nurses, and patients along with customer-based behavior have a significant effect on the patient’s satisfaction and service quality promotion [45–48]. Interpretation of these results has shown that the shortages of health workforces, insufficient salaries and payments, cancelation of the leaves, and other challenges related to the health workforces during a pandemic can, directly and indirectly, affect their attempts in effective communicating with the patients.

This study confirmed that the patients view the quality of the services at an average level. The previous study showed different results when investigating the patients’ perception of the quality of the services. A study on outpatients during the COVID-19 indicated that participants considered the service quality acceptable [54]. It is mainly because outpatients need less time to get the services they need. Another study in a military hospital further showed that COVID-19 patients were satisfied with all levels of services; this may tie back to the premise that these kinds of hospitals were fully equipped with modern facilities [39]. It is well discussed that hospitals services faced serious challenges during COVID-19; this may affect negatively services quality [99, 100]. This fact may the reason why our participants did not show a positive view of the services they received. Martinez et al. concluded that the patients who were admitted to the intensive care unit before the pandemic was satisfied with all aspects of the service quality [55]. A meta-analysis confirmed that before the pandemic the quality of services stated by the Iranian patients was placed at an acceptable level compared to other countries [56]. In general, prior research reported varied results in Iran [57–61]. Such difference findings may be related to study settings, the time of the study, sample size, and the participants’ status in terms of socio-economics factors.

The other results of the present study showed that in a service quality dimension, 37.05% of the old patients have evaluated the “Tangibles” dimension at a good level. Similarly, Hashemi et al. in Kerman, Iran [78], Ahmadi et al. in Jahrom, Iran [62], and Zarei et al. [63] have declared that among all dimensions of quality, “Tangibles” have a better status. In contrast with the present study, Khezeli et al. with the aim of evaluating the quality gap in hospital services from the COVID-19 patients’ viewpoints in Tehran have indicated that the lowest perceived mean of quality services was related to the “Tangibles” dimension [57]. Similarly, Goula et al. in Greece have also reported the level of service quality in all 5 dimensions less than the patients’ expectations, and the most negative gap was related to the “Tangibles” dimension [81]. In a qualitative study in China, most of the interviewees have announced that appropriate facilities and equipment in the hospital departments may lead to promoting the patients’ spirit [79]. Farajzadeh et al. in Tehran have also introduced some factors as the main effective determinants of COVID-19 patients’ satisfaction. Among them, we can refer to modern facilitations and medical equipment as well as welfare services [39]. Appropriate physical conditions not also lead to comfort among service receivers but also result in comfort in the work environment and finally cause the better condition of service delivery. The present participants have evaluated the “Assurance” dimension at a weak level (22.28%). Similarly, Goula et al. have announced that the two dimensions of “Tangibles “ and “Assurance” have the most negative gap respectively [81]. According to Sina et al. the “Assurance” dimension was the inappropriate status [64].

The present results have shown that there is a statistical correlation between responsiveness and service quality from the old patients’ perspectives. In this line, in research, authors found that responsiveness positively influences the patient’s satisfaction. Using multi-variable analysis, they confirmed that inpatients’ satisfaction was positively related to three aspects of responsiveness such as quick attention, transparent relationship, and dignity [35]. In another study in Nigeria, it was also found that patients’ satisfaction is positively correlated with quick attention and transparent relationship [38]. Similarly, such findings were further approved by the research conducted in Pakistan [34]. In this regard, it seems that more responsiveness of the hospitals and the healthcare workers can lead to a higher perceived service quality from the patients. So, health care policymakers should try to find applied mechanisms to consider all the determinants influencing the responsiveness and the service quality with a systemic approach.

According to the results, the responsiveness level from the older adults’ perspective was significantly different based on gender and marital status. On this basis, the status of responsiveness was highly reported by the male and married old patients. Ameryoun et al. have similarly achieved higher means of service quality among married participants [77]. Such results need to be deeply investigated to explore more relations between other probable demographic variables and the quality of services particularly among older adults.

Considering the present results, the quality of services from the old patients’ viewpoints was subject to gender and level of education so that the higher mean of service quality was observed from the men and the old patients with lower education. Similarly, Hashemi et al. in Kerman, Iran have shown that the mean of service quality in an educational hospital was higher in male patients than the female [78]. Gonzalez-Valentin et al. have also reported higher satisfaction and more appropriate viewpoints toward service delivery in the male participants [80]. In another study in 15 Chinese hospitals, the female participants’ attention to service quality and the hospital environment was higher than the men. Moreover, the women have more claims against the inappropriate environment of the hospitals in comparison with the male patients [79]. It seems that different characteristics related to gender can lead to more sensitivity of the women toward the physical environment in public places than the men. At the same time, women have higher expectations and dissatisfaction and as a result have a poorer evaluation of the service quality.

Regarding the relationship between perceived quality of services and level of education, similar studies in Iran show that those people with a lower level of education have evaluated the service quality at a higher level at the same time, level of dissatisfaction was higher among people with higher education or Diploma [78, 82]. Goula et al. have shown that people with a lower level of education have lower expectations. They have also benefited from more satisfaction in the areas of the patient-physician relationship and hospital facilities and equipment [81]. Wudu in Ethiopia has indicated that having an elementary level of education can be considered as an important predictor of patient satisfaction from nursing cares in the hospitals [83]. According to Bakar et al. level of education is considered as an effective factor in forming the patients` expectations [84]. This result can be justified in a way that people with a higher level of education have more social relationships and more access to information resources. They are also more familiar with their rights at the time of receiving services. So the shortages and failures of the hospitals become more highlighted for them and their satisfaction shows a significant decrease.

Theoretically, our finding shed light new insights on the utility theory by adding new evidence as to how the older patients view the quality of health services and how important is human rights in healthcare systems. The results of this study further lead to the awareness of policymakers and hospital managers about the responsiveness and quality of services. By identifying the dimensions that had a low score and planning to strengthen these dimensions, the necessary conditions are supposed to improve the responsiveness to non-medical needs., leading to increasing the quality of services and ultimately patients satisfaction. In addition, considering the most important aspects of responsiveness affecting the quality of services, the priorities for action in this field can be examined.

## Limitations

This study has some limitations from which we can refer to its cross-sectional nature that restricts the possibility of determining the casualty relationships. So conducting the longitudinal studies with different methods of data collection was recommended. Meanwhile, designing the qualitative studies with the aim of exploring the in depth experiences of the old people from hospital service delivery and responsiveness is emphasized.

## Conclusion

According to the results, responsiveness and quality services were reported at the average level. There was a positive significant correlation between these two variables. Such a finding emphasized the necessity of attention to applied and effective solutions to increase the responsiveness level and the service quality. At the same time, different dimensions of responsiveness were recognized as the predictors of service quality. This finding indicates that improvement in each of the responsiveness dimensions can lead to making a better perception of service quality from the older adults’ viewpoints.

## Data Availability

Whilst identifying/confidential patient data should not be published within the manuscript, the datasets used and/or analyzed during the current study are available from the corresponding author on reasonable request.
